# Signal Transduction Alterations in Glioma: Implications for Diagnosis and Therapy

**DOI:** 10.1155/2012/704247

**Published:** 2012-12-09

**Authors:** Laura Cerchia, Juan-Carlos Martinez Montero, Parisa Monfared

**Affiliations:** ^1^Istituto per l'Endocrinologia e l'Oncologia Sperimentale “G. Salvatore” (IEOS), CNR, 80131 Naples, Italy; ^2^Anatomia Patologica, Instituto Oftalmico, Hospital Universitario Gregorio Marañón, 28007 Madrid, Spain; ^3^European Insitute for Molecular Imaging (EIMI), Wilhelms University Muenster, 48149 Nünster, Germany

Malignant gliomas are the leading cause of central-nervous-system-tumour-related death, and despite recent advances in surgery, radiotherapy, and chemotherapy, current treatment regimens have a modest survival benefit; the prognosis is even worse in children with brain stem malignant gliomas.

Gliomas are divided into four clinical grades on the basis of their histology and prognosis. The most malignant grade 4 astrocytoma, or glioblastoma multiforme (GBM), either arises *de novo *(usually associated to epidermal growth factor receptor pathway activation and PTEN inactivation leading to PI3K kinase/AKT activation pathway) or progresses from lower grade to higher grade over time (characteristically due to p53 and retinoblastoma pathways inactivation). Due to a combination of its complex phenotype and organ-specific clinical manifestations, efforts to refine GBM treatment with targeted therapies have largely been frustrated. Therefore, the identification and characterization of signal transduction pathways alterations, with a pathogenic role on glioma development and progression, may contribute to the identification of therapeutic targets aimed at a more efficient treatment.

The seven tightly organized papers in this special issue give an update of all the latest concepts about the molecular mechanisms of pathogenesis of glioblastoma and new therapeutic opportunities.

The molecular characteristics of angiogenesis, a key event for glioma survival, aggressiveness and growth, are addressed by two well-balanced papers. S. Bulnes et al. review angiogenic signaling altered in a rat glioma model and discuss on the selection mechanisms for more aggressive subpopulation with invasive phenotype. They show that glioma stem cells and vascular endothelial cells play a relevant role in the angiogenic process, and referring to molecular pathways, hypoxia inducible factor-1 and vascular endothelial growth factor are the most significant. The papers by V. Cea et al. offers an overview of the most relevant issues about antiangiogenic therapy for glioma, presenting several available drugs that are used or can potentially be utilized for the inhibition of angiogenesis in glioma, focusing on the key mediators of the molecular mechanisms underlying the resistance of glioma to anti-angiogenic therapy.

Two interesting and novel papers discuss epigenetic mechanisms producing signal pathways deregulation in gliomas. The paper by R. Alelù-Paz et al. is a nice addition to the current literature about epigenetic changes in human cancer, particularly in gliomas. The emerging role of cancer stem cells in the pathophysiology of cancer is as well discussed. R. Martinez has written a paper describing epigenetic and genetic alterations in gliomas, resulting in deregulation or functional disruption of tumor suppressor and oncogenes. In both papers, the discussion of epigenetic alterations in the pathogenesis and evolution of gliomas clearly indicate their crucial function for discovering new biomarkers for detection and prognosis and for development of new pharmacological strategies.

L. Catacuzzeno et al. clearly introduce the reader to the structural, biophysical, pharmacological, and modulatory properties of the intermediate conductance calcium-activated K (KCa3.1) channels. They describe the importance of the KCa3.1 channels in glioblastoma cell functions. These channels are highly expressed in glioblastoma cells if compared to the normal brain parenchyma and play an important role in the control of glioblastoma cell migration, a critical process that represents major causes for tumor progression and for recurrence following tumor surgical resection. Altogether, data suggest KCa3.1 channels as potential candidates for a targeted therapy against glioma.

The research paper by H. L. Watt et al. evaluates the biological responses of glioma cells to combined treatment with RTK inhibitors, DNA damaging agents, and octreotide, an agonist of the somatotropin receptor. Changes in the activation profile of EGFR mitogenic signaling and DNA damage response pathway, as well as apoptosis and cell cycle distribution were analyzed. The results support the notion that the effects of combined therapy on glioma cells mostly depend on the specific context of cell cycle arrest.

A crucial challenge for human glioma treatment is to deliver drugs effectively to invasive glioma cells residing in a sanctuary within the central nervous system. S. Catuogno et al. discuss recent results on the use of oligonucleotides that will hopefully provide new effective treatment for gliomas. Oligonucleotide-based approaches, including antisense, microRNAs, small interfering RNAs, and nucleic acid aptamers, look very promising particularly to overcome challenges presented by the blood-brain barrier.

In total, we hope that these contributions will provide a well-rounded overview of histopathology, molecular biology, and current treatment strategies for glioma.

